# Supplementary material for: Could a Non-Cellular Molecular Interactome in the Blood Circulation Influence Pathogens’ Infectivity?
Source: Cells. 2023 Jun 23;12(13):1699. doi: 10.3390/cells12131699 (PMC10341357; doi:10.3390/cells12131699)
Supplement: Supplementary file 1 [file cells-12-01699-s001.zip › cells-2416650-supplementary.pdf]

Electronic Supplementary Information

Could a Non-Cellular Molecular Interactome in the Blood Circulation Influence Pathogens' Infectivity?

Eugenio Hardy [1,*], Hassan Sarker [2] and Carlos Fernandez-Patron [2,*]

[1] Center of Molecular Immunology, P.O. Box 16040, Havana 11600, Cuba.
[2] Department of Biochemistry, Faculty of Medicine and Dentistry, College of Health Sciences, University of Alberta, Edmonton, AB T6G 2H7, Canada; hsarker@ualberta.ca
* Correspondence: eugenio@cim.sld.cu (E.H.); cf2@ualberta.ca (C.F.-P.)

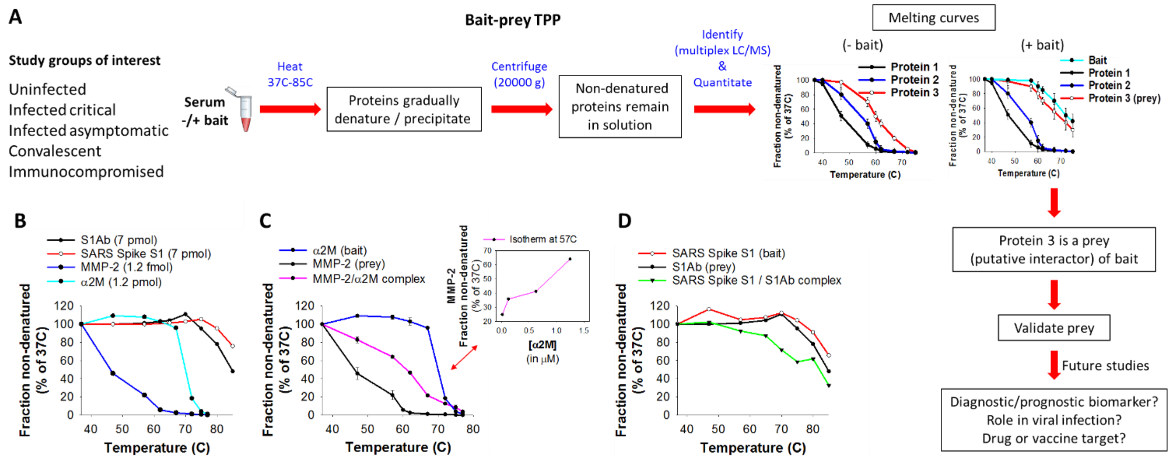

Supplementary Figure S1. Illustration of a simple proteomic strategy (termed herein bait-prey thermal proteome profiling or "bait-prey TPP") that could be applicable to characterize the interactome of components of a pathogen. (A): Principle of bait-prey-TPP-approach. Proteins generally denature and subsequently precipitate when subjected to heat. Upon interaction with other molecules (such as metabolites, peptides and proteins), the temperature at which denaturation occurs may change. Interacting proteins co-precipitate upon heat-induced denaturation and exhibit similar solubility across different temperatures [1]. Protein mixtures are typically heated for 10 min at temperatures ranging from 37°C (at which all proteins are soluble) to 85°C (at which most proteins denature and precipitate). At each temperature point, the non-denatured proteins remaining in the soluble fraction can be identified and quantified using unbiased protein detection techniques (e.g., liquid chromatography-mass spectrometry [LC-MS]; attomole level sensitivity) and targeted techniques (e.g., Western immunoblotting, ELISA; low femtomole level sensitivity). The results can be documented as a melting curve: 'y'-axis indicates the amount of thermally stable protein (i.e., non-denatured, not precipitated, soluble); 'x'-axis indicates the temperature range. The TPP melting curves can be compared by ANOVA or (dis-) similarity measures, such as Euclidean distance [2]. **(B): Proof-of-principle that TPP can resolve proteins with different thermal stability**. Melting curves for SARS-CoV-2 Spike S1 Antibody (S1Ab), SARS Spike protein S1, matrix metalloproteinase (MMP-2), and a2-

macroglobulin (α2M) are shown. Protein amounts (in parentheses) hint at the high sensitivity of TPP. **(C): Proof-of-principle showing how TPP can detect interactors of bait proteins**. In blood, α2M is a well-known interactor and inhibitor of MMP-2. TPP shows a shift in the melting curves of MMP-2 (12 nM) in the presence of α2M (1.25 µM) compared to control (vehicle). The isothermal concentration-response analysis indicates that the higher the concentration of α2M, the more MMP-2 is found in complex with α2M in the soluble fraction, suggesting that α2M could be used as bait for MMP-2. **(D): Proof-of-principle showing how bait-prey TPP can detect interactors of S1 proteins**. TPP shows a shift in the melting curves of SARS Spike protein S1 in the presence of S1Ab compared to control (vehicle), suggesting an interaction between them. B-D strongly suggests that bait-prey TPP could be applied to rapidly identify and/or validate potential SARS-CoV-2 interactors (prey) in blood circulation using recombinant SARS-CoV-2 proteins as bait. It is important to exclude false-positive interactors and identify only positive S1-specific interactors, for which isothermal concentration-response experiments should be carried out. This can be complemented by parallel validation conducted using Ni-beads- and S1 antibody-based pull-down assays to isolate S1 with interactors using the soluble fraction at each temperature of the thermal stability assay and the mixture of S1 and blood sample. Detection of S1 and the identification of candidate S1 interactors may be performed by Western-immunoblotting (S1Ab) and LC-MS, respectively. Data analysis can be aided by bioinformatics tools described before by us [3].

References

[1] C.S.H. Tan, K.D. Go, X. Bisteau, L. Dai, C.H. Yong, N. Prabhu, M.B. Ozturk, Y.T. Lim, L. Sreekumar, J. Lengqvist, V. Tergaonkar, P. Kaldis, R.M. Sobota, P. Nordlund, Thermal proximity coaggregation for system-wide profiling of protein complex dynamics in cells, Science. 359 (2018) 1170–1177. https://doi.org/10.1126/SCIENCE.AAN0346.

[2] L. Dai, N. Prabhu, L.Y. Yu, S. Bacanu, A.D. Ramos, P. Nordlund, Horizontal Cell Biology: Monitoring Global Changes of Protein Interaction States with the Proteome-Wide Cellular Thermal Shift Assay (CETSA), Annu Rev Biochem. 88 (2019) 383–408. https://doi.org/10.1146/ANNUREV-BIOCHEM-062917-012837.

[3] J. Teitz, J. Sanders, H. Sarker, C. Fernandez-Patron, Potential of dissimilarity measure-based computation of protein thermal stability data for determining protein interactions, Brief Bioinform. (2023); bbad143. https://doi.org/10.1093/bib/bbad143
